# Low-dose of phenolic rich extract from *Annona squamosa* Linn leaves ameliorates insulin sensitivity and reduces body weight gain in HF diet-induced obesity

**DOI:** 10.3389/fnut.2023.1146021

**Published:** 2023-07-19

**Authors:** Hana Alkhalidy, Anas Al-Nabulsi, Reham Mhawish, Dongmin Liu

**Affiliations:** ^1^Department of Nutrition and Food Technology, College of Agriculture, Jordan University of Science and Technology, Irbid, Jordan; ^2^Department of Human Nutrition, Foods and Exercise, College of Agricultural and Life Sciences, Virginia Tech, Blacksburg, VA, United States

**Keywords:** obesity, diabetes, insulin resistance, *Annona squamosa* Linn, adiposity

## Abstract

Obesity is associated with metabolic abnormalities that increase the risk and severity of several diseases. This study aimed to explore whether the aqueous extract of *Annona squamosa* Linn leaves (ASE) can ameliorate metabolic abnormalities associated with high fat (HF) diet-induced obesity. Forty-eight male Wistar rats were distributed among four treatment groups: a standard low-fat diet group, a HF diet group, and two HF diet groups with a daily oral dose of ASE (100 or 200 mg/kg body weights) administered for 9 weeks. Daily energy intake, body weight, blood glucose levels and glucose tolerance, and insulin tolerance were evaluated. At the end of the study, organs, and tissues were collected and weighed for analysis, and blood samples were collected to determine the serum insulin levels and serum liver enzymes. Total phenolic and flavonoid contents and 2,2-Diphenyl-1-picrylhydrazyl free radical antioxidant activity of the ASE were evaluated. Oral administration of the low dose of ASE to HF diet-fed rats significantly reduced the long-term food intake and body weight gain without altering adiposity compared with untreated HF diet-fed rats. This outcome was accompanied by a significant improvement in insulin sensitivity and a reduction in fasting blood glucose (FBG) levels measured at weeks 6 and 9 of the study. The high dose of ASE had a short-term effect on body weight gain and food and caloric intake, and in the long-term, it improved FBG levels measured at weeks 6 and 9 of the study. The high dose of ASE resulted in hyperinsulinemia and high homeostatic model assessment for insulin resistance (HOMA-IR) value compared to healthy rats. Total phenolic and flavonoid contents were 74.9 ± 0.491 mg of gallic acid equivalent and 20.0 ± 0.091 mg quercetin equivalent per g of ASE, respectively. The antioxidant activity of ASE expressed as half-maximal inhibitory concentration (IC_50_) value was 8.43 ± 0.825 mg/mL. These data suggest that ASE can safely and potently reduce the development of insulin resistance induced by HF diet feeding and lowering body weight gain in a dose-dependent manner.

## 1. Introduction

In recent decades, obesity has received considerable attention as a major health problem. Obesity is often defined as a condition of abnormal or excessive fat accumulation in adipose tissue, to such an extent that health may be adversely affected (1). Obesity is associated with a range of comorbidities; it is now considered a major contributor to the global burden of disease and disability such as sleep apnea (2), asthma (3), stroke, congestive heart failure, ischemic heart disease (4), and cancer (5). Besides, obesity is a leading pathogenic factor for developing insulin resistance (IR), which is one of the most powerful predictors of future progression to type 2 diabetes mellitus (T2DM) (6).

IR refers to the inability of the target cells to respond to the effects of insulin on glucose uptake, metabolism, or storage (7). There is a strong relationship between obesity and IR that could be explained by the increase in inflammatory cytokines production in obesity such as tumor necrosis factor-α, and interleukin-6 (8). The increased systematic levels of these cytokines may inhibit insulin signaling downstream of the insulin receptor (8). Also, dietary fats and more specifically excess intake of saturated fatty acids (SFAs) are strongly linked to the development of obesity and IR, as demonstrated in numerous studies using experimental animal models (9) and in humans (10). The SFAs may change membrane lipid composition and consequently impair its fluidity, leading to impaired transport and binding functions of the membrane that result in the reduced overall metabolic rate of the cells (11). Also, a decrease in the rate of the beta-oxidation process for SFAs makes them poorly used for energy and more likely to be stored in the adipose tissue (12). Moreover, the SFAs were shown to enhance adipocyte growth and development (13).

There is a growing interest in plants traditionally used to treat obesity (10) and IR because of their effectiveness, minimal side effects in clinical experience, and relatively low cost (14). *Annona squamosa* Linn a multipurpose tree with edible fruit that is commonly known as custard apple, coming from the *Annonaceae* family specially Annona genera. It has been reported that *Annona squamosa* Linn elicits a number of health benefits, including antitumor (15), and antioxidant activities (16) of the fruit part, and antifungal (17), and antimicrobial activities (18) of the leaves. These beneficial effects may be due to its content of several bioactive compounds such as glycosides, phenols (17), flavonoids (19), and alkaloids (16).

Studies have shown promising outcomes on the effect of *Annona squamosa* Linn leaves on experimental diabetic animals. For example, the ethanolic extract of *Annona squamosa* Linn leaves improved glucose tolerance, decreased low-density lipoprotein (LDL), triglycerides (TG), and total cholesterol (TC) levels in streptozotocin (STZ)-induced diabetic rats and alloxan-induced diabetic rabbits (19). In addition, a glycosylated flavonoid (quercetin-3-O-glucoside) isolated from *Annona squamosa* Linn leaves by methanolic extraction was showed the antidiabetic effect in alloxan-induced diabetic rats, which was associated with improved insulin and antioxidant status (20). The hot-water extract of *Annona squamosa* Linn leaves showed the most favorable effect on glucose control. A dose of 350 mg/kg body weight significantly improved glucose tolerance in an acute period of 1 h and 2 h after administration in alloxan-induced diabetic rabbits and STZ-induced diabetic rats. Moreover, 15 days of administration of the aqueous extract decreased the levels of TC, LDL, TG, glycosylated hemoglobin level, and increased high-density lipoprotein (HDL), TC, pancreatic serum insulin, and glucose uptake in the muscle tissues (19). Similarly, the aqueous leave extract has been reported to significantly reduce blood glucose and improve insulin activity in STZ-induced diabetic rats when given a dose of 300 mg/kg body weight for 30 days (21). A recent study investigated the effect of hot-water extract (250 mg/5 mL/kg) on high-fat (HF) fed obese rats and the 9 days of treatment showed improvement in glucose tolerance and plasma insulin levels with minimal effect on energy intake, and body weight (22). Most of the studies described above have investigated the anti-diabetic effects of *Annona squamosa* Linn in chemically induced diabetes (alloxan- or STZ-induced diabetic animals) or investigated the anti-obesity effect after inducing obesity in animal models and in the short-term. However, studies about the long-term effect of the leave extract of this plant on IR and obesity have not been reported previously. Therefore, the aim of our present study was to assess the long-term preventive effects of a hot-water extract of *Annona squamosa* Linn leaves on obesity and diabetes in a HF-diet-induced obesity and IR animal model.

## 2. Materials and methods

### 2.1. Plant leaves collection and extract preparation

Leaves of the plant *Annona squamosa* Linn were collected from the Jordan Valley. The plant was identified by Dr. Mohammad Al-Gharaibeh from the Plant Production Department at Jordan University of Science and Technology. A voucher specimen (PHS-135) was deposited in the Herbarium of the Faculty of Pharmacy at Jordan University of Science and Technology. The fresh leaves were weighed and washed with cold tap water and then rinsed with cold distilled water. Washed leaves were placed on towel paper to absorb the excess water and were dried at room temperature for 5 days in the absence of sunlight. The leaves were evenly spread on a surface coated by aluminum foil paper or on a metal tray. Leaves were cut into small pieces and pulverized in a mechanical blender and the powdered plant material was used for the preparation of the aqueous extract.

Distilled water was added to the powder with a ratio of 1:20 (weight/volume) and subjected to boiling. The resulting dark-brown extract was cooled and filtered. The filtrate was centrifuged at 10,000 rpm at room temperature for 10 min and the sediment was discarded. The concentrated crude extract was lyophilized and stored in sterile bottles at −20°C for further use.

### 2.2. Animal study

Forty-eight male Wistar rats weighing between 160 and 180 g were used in this study. The animals were obtained from and kept at the animal house at Jordan University of Science and Technology. The animals were kept under standard laboratory conditions (12 h light: 12 h dark and under constant room temperature during the experimentation period with water and food available at all times). Rats were given a week to acclimate before the beginning of the experiment. The rats were divided into 4 groups (*n* = 12 rats/group) with blood glucose [fasting (FBG) and nonfasting (NFBG)] and body weight balanced. The first group of rats was fed a standard chow diet, with approximately 5% of calories derived from fat, 17% from protein, and 78% from carbohydrates. The second, third, and fourth groups were fed HF-diet with 60% of the calories from fat (Supplementary Table S1; Research Diets Inc., New Brunswick, NJ, United States). The third and fourth groups were given, at the beginning of the HF-diet feeding, a daily dose of ASE; 100 (low dose) or 200 (high dose) mg/kg body weight administered orally. The first (LF) and second (HF) groups received equivalent volumes of the vehicle (2% 2-methyl cellulose). The study protocol and procedures performed in this study were reviewed and approved by the Animal Care and Use Committee at the Jordan University of Science and Technology (362-2018).

#### 2.2.1. Metabolic studies

##### 2.2.1.1. Body weight, food intake, and caloric intake measurements

Rats’ body weight and food intake (FI) were recorded weekly throughout the study. Caloric intake was calculated by multiplying the calories/g diet (LF or HF diet) by the grams of diet consumed per rat per day.

##### 2.2.1.2. Blood glucose control and tolerance measurements

The FBG and NFBG concentrations in the tail vein blood samples were measured using a glucometer (The ACCU-CHEK, United States) at the beginning of the experiment and then examined every 3 weeks at 3, 6, and 9 weeks throughout the study. FBG was measured after 16 h food withdrawal and after 2 days from NFBG. The oral glucose tolerance test (OGTT) was performed at the end of the eighth week. The rats fasted in the morning at the beginning of the light cycle for 6 h and glucose was administered orally with a single dose (1.50 g/kg body weight). The blood samples were collected from the tail vein and glucose levels were measured using a glucometer at times 0, 15, 30, 60, and 120 min of glucose administration. Total glycemic responses to OGTT were calculated from respective areas under the curve (AUC) of glycemia during the 120 min observation period using the trapezoidal rule (23).

##### 2.2.1.3. Insulin tolerance

An insulin tolerance test (ITT) was performed with the same rats 2 days after the OGTT. The rats were fasted for 6 h and then injected intraperitoneally (IP) with a single dose of human insulin (0.75 IU/kg body weight), and blood glucose was measured before and at 15, 30, 60, and 120 min after insulin administration. The AUC was calculated using the trapezoidal rule (23).

#### 2.2.2. Blood parameters

At the end of the experiment, the rats were fasted overnight (14 to 16 h) and euthanized using a single intraperitoneal injection of ketamine and xylaxine (90 and 10 mg/kg body weight, respectively) that was freshly prepared. Blood samples were collected by cardiac puncture and centrifuged at 15,000 rpm for 10 min. The serum was collected carefully by pipetting and stored at −20°C for further analysis. Serum insulin levels were determined using a rat insulin ELISA kit (Mercodia, Sweden). The homeostatic model assessment for IR (HOMA-IR) was calculated from fasting insulin and glucose values by using Matthew’s formula (24). The serum enzymes, alanine aminotransferase (ALT), aspartate aminotransferase (AST) were analyzed by enzymatic methods using commercially available kits (BioSystems S.A, Spain).

#### 2.2.3. Organs weighing and fat tissue

The body fat such as subcutaneous pads (inguinal) and several visceral pads (epididymal, retroperitoneal fat pads located on the kidneys and mesenteric) was isolated, weighed, and then normalized to the body weight. In addition, multiple organs were isolated and weighed including the liver, heart, kidney, pancreas, and spleen. The livers were homogenized and extracted with chloroform: methanol (2:1, v/v) (25), and the total lipid content was measured.

### 2.3. Determination of the total amounts of antioxidant compounds

#### 2.3.1. Total phenolic content

The total phenolic content (TPC) in the extract was determined as previously described by Tomar and Sisodia with some modifications (26). 100 uL of Folin–Ciocalteu reagent (tenfold diluted) was added to 20 uL of the extract (5 mg/mL) and mixed. Then, 80 uL of 7.5% Sodium Carbonate was added to the mixture. The sample was incubated in the dark for 1 h at room temperature. The absorbance was recorded at 760 nm using a microplate reader. A standard curve of different concentrations of gallic acid (0.06–0.2 mg/mL) was used to determine the total phenolic content of the extract. Measurements were taken in triplicate. The results were expressed as milligram gallic acid equivalent (GAE) per gram of the dry extract.

#### 2.3.2. Total flavonoid content

The total flavonoid content (TFC) in the extract was determined as previously described by Aryal et al. (27) with some modifications. 6 uL of 10% Aluminum Chloride was added to 30 uL of the extract (5 mg/mL) and mixed. Then, 6 uL of 1 M Potassium Acetate was added to the mixture. Finally, 168 uL of distilled water was added. The sample was incubated in the dark for 30–45 min at room temperature. The absorbance was recorded at 415 nm using a microplate reader. A standard curve of different concentrations of quercetin (0.06–0.2 mg/mL) was used to determine the total flavonoid content of the extract. Measurements were taken in triplicate. The results were expressed as milligram quercetin equivalent (QE) per gram of the dry extract.

#### 2.3.3. Determination of antioxidant activity

The antioxidant activity of the ASE was measured using the 2,2-Diphenyl-1-picrylhydrazyl (DPPH) free radical antioxidant activity, as described by Mahdi-Pour et al. (28) with some modifications. In every 2 uL of each extract concentration (0.3–10 mg/mL); a 200 uL of 0.004% DPPH in methanol was added. The mixtures were incubated in the dark for 30 min at room temperature. The absorbance was recorded at 517 nm using a microplate reader. Butylated Hydroxytoluene (BHT) was used as a standard antioxidant. Measurements were taken in duplicate. The results were reported as half-maximal inhibitory concentration (IC_50_) values, the higher the number the lower is the antioxidant activity.

### 2.4. Statistical analysis

The data were analyzed by one-way analysis of variance using JMP® Pro 11.0.0 (2013, United States). If significant differences between treatments (*p* < 0.05) were observed, Tukey’s range test was then performed for pairwise comparisons. Values are expressed as the mean ± standard error of the mean.

## 3. Results

### 3.1. Low-dose ASE reduced HF-diet-induced body weight gain, food, and caloric intake

**Figure 1:**
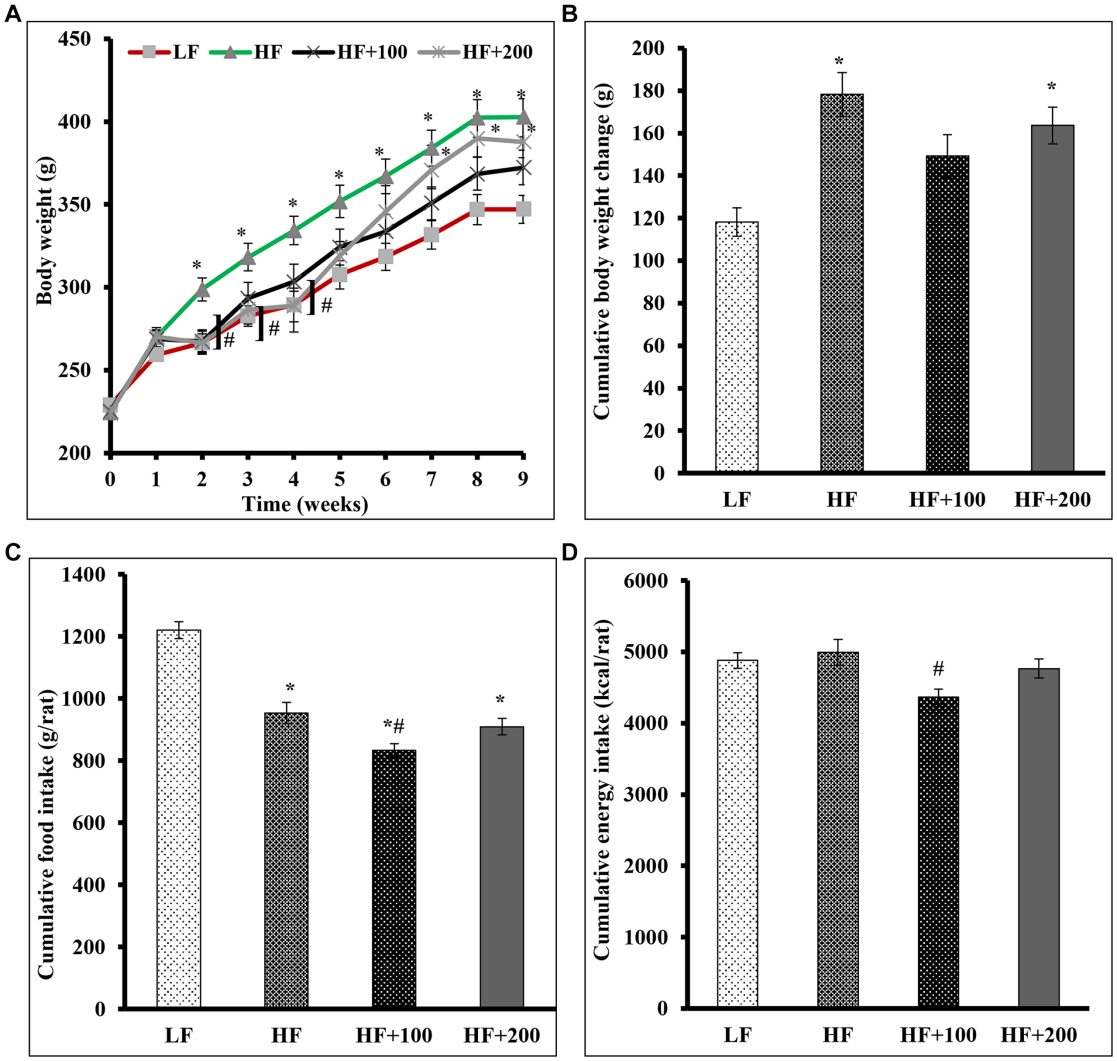
Low-dose of ASE treatment reduced HF diet-induced body weight gain, cumulative food consumption and cumulative energy consumption. **(A)** Body weights were measured weekly. **(B)** The cumulative weight change was the cumulative difference between week 0 and other weeks in grams. **(C)** The cumulative average food daily intake was calculated on a weekly basis. **(D)** The cumulative energy consumption (kcal) was calculated on a weekly basis. Data are shown as means ± SEM (*n* = 11–12). ^*^p < 0.05 vs. LF group; ^#^*p* < 0.05 vs. HF group. LF, low-fat diet-fed rats; HF, high-fat diet-fed rats; HF + 100, high-fat diet-fed rats treated with low dose ASE (100 mg/kg body weight); HF + 200, high-fat diet-fed rats treated with high dose ASE (200 mg/kg body weight).

This study was undertaken to test whether ASE (low dose; 100 mg/kg body weight, or high dose; 200 mg/kg body weight) is capable of preventing or ameliorating IR associated with obesity in rats receiving HF diet and treated with ASE or vehicle for 9 weeks. In this study, rats in the HF group were significantly heavier than that of the LF group at the end of the second week and throughout the study period (Figure 1A). Treatment with the low dose of ASE started to significantly reduce the gain in body weight from the second week of the study and continued to reduce it to the end of the study. However, the high dose of ASE lowered body weight gain that continued only until the end of the sixth week of treatment. The weekly change in body weight for rats is shown in Supplementary Figure S1A showing the same trend in body weight.

After 9 weeks, the HF diet increased the cumulative average body weight change (178.3 ± 35.9) as compared with those that consumed the LF diet (118.2 ± 22.9; Figure 1B). Our finding suggests that the ASE might act more efficiently at low doses on the cumulative average weight change (149.2 ± 33.5) than at the high dose of ASE (163.6 ± 29.9).

The daily FI is shown in Supplementary Figure S1B and the calculated cumulative FI at the end of the study showed that rats in the HF diet group had lower cumulative FI (953.3 ± 34.4) as compared with that of those consumed the LF diet (1220.3 ± 27.5; Figure 1C). The low dose of ASE significantly lowered the cumulative average daily FI (833.9 ± 21.2) compared to the other groups. The cumulative FI for rats treated with the high dose of ASE did not differ (909.7 ± 25.9) from the cumulative FI for rats fed the HF diet (953.3 ± 34.4).

The daily energy intake is shown in Supplementary Figure S1C and at the end of the study the cumulative energy intake did not differ among the HF (4995.1 ± 180.4), LF (4881.2 ± 110.1), and the high dose of ASE groups (4767.0 ± 136.1; Figure 1D). While the cumulative energy intake was significantly lower in the low dose of ASE (4369.5 ± 111.1) compared to the HF group.

### 3.2. ASE reduced the HF diet effect on FBG without affecting glucose tolerance

**Figure 2:**
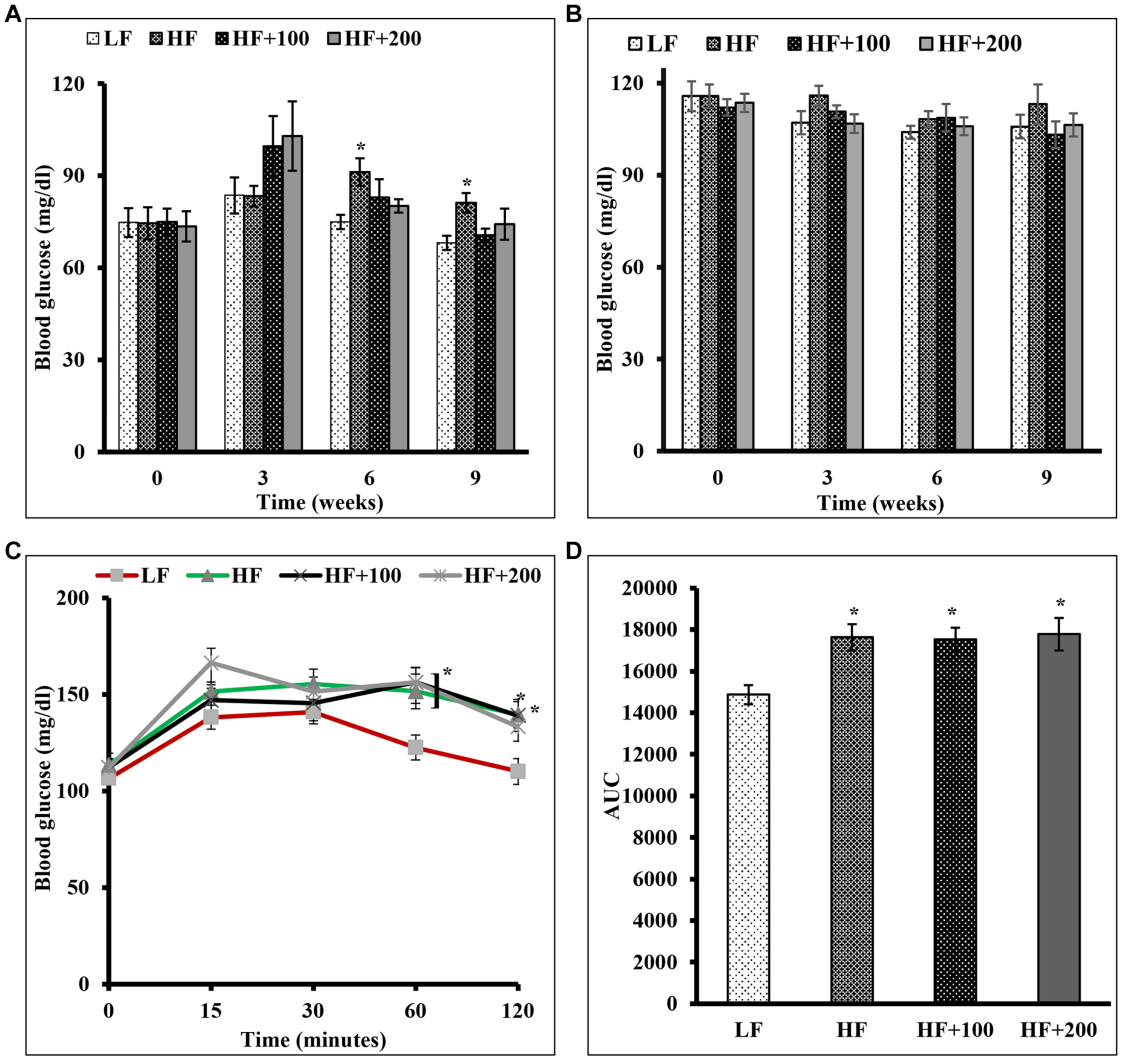
Oral administration of ASE improved fasting blood glucose in HF diet-fed rats, but had no influence on nonfasting blood glucose and the glucose intolerance in rats fed the HF diet. **(A)** Fasting and **(B)** non-fasting blood glucose levels were measured at indicated time points during 9 weeks of ASE treatment every 3 weeks throughout the study **(C)** Oral glucose tolerance test (OGTT) was performed as described in the Materials and Methods section at the end of week 8 of the study. **(D)** The area under the curve (AUC) was calculated for OGTT. Data are shown as means ± SEM (*n* = 11–12). ^*^p < 0.05 vs. LF group. LF, low-fat diet-fed rats; HF, high-fat diet-fed rats; HF + 100, high fat diet-fed rats treated with low dose ASE (100 mg/kg body weight); HF + 200, high-fat diet-fed rats treated with high dose ASE (200 mg/kg body weight).

The effect of ASE on glucose control was evaluated by measuring FBG, NFBG levels, and performing a glucose tolerance test. FBG and NFBG levels were evaluated before the beginning of the treatment and every 3 weeks. At week 6 of the study, rats fed the HF diet displayed significantly elevated FBG compared to rats in LF groups, and this elevation continued to be significant until the end of the study. The oral administration of ASE reduced the HF diet-induced rise in FBG level at week 6 of the study (Figure 2A). And at the end of study, rats treated with ASE (100 and 200 mg/kg) continued to have lower FBG levels (70.5 ± 2.3 mg/dL and 74.2 ± 5.07 mg/dL, respectively) as compared to HF diet-fed rats (81.2 ± 3.2 mg/dL) and this effect on FBG was not significant. Treatment with ASE reduced FBG to levels closer to the levels in the LF group (68.2 ± 2.3 mg/dL). There were no differences in NFBG levels between groups (Figure 2B).

An OGTT was performed at the end of the eighth week. The results showed that the difference in glucose tolerance for all groups was not significant in the first 30 min after the oral glucose challenge. After 1 h of glucose administration, the ASE-treated groups had significantly elevated blood glucose as compared with the LF group, with no significant differences between the HF and LF groups. After 2 h, all rats except rats fed the high-dose of ASE had significantly elevated blood glucose as compared with LF diet-fed rats (Figure 2C). The calculated AUC (Figure 2D) for OGTT blood glucose levels showed that rats treated with HF diet and ASE had significantly higher AUC than LF diet-fed rats.

### 3.3. Low-dose ASE improved insulin sensitivity

**Figure 3:**
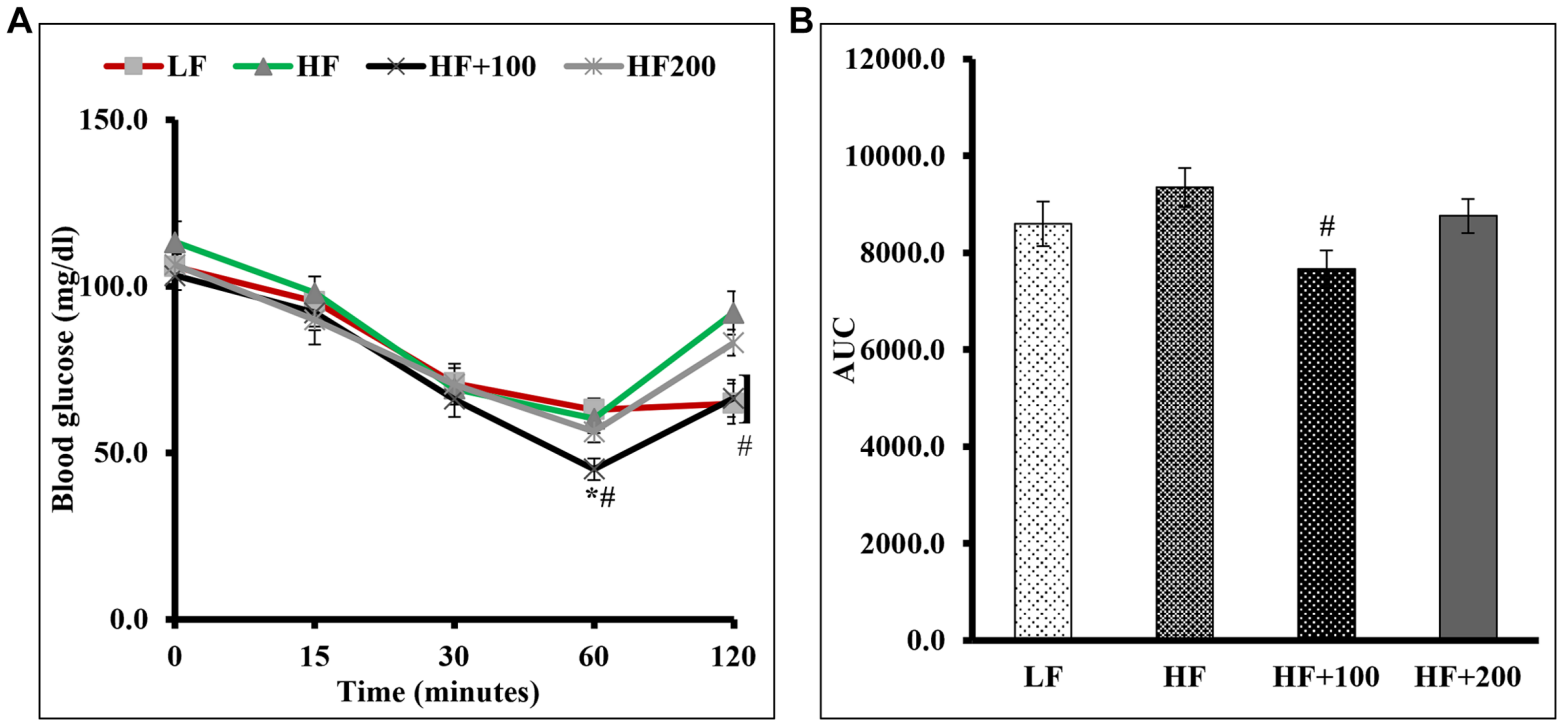
Low dose of ASE treatment improved insulin sensitivity in rats fed the HF diet. **(A)** Insulin tolerance test (ITT) was performed as described in the Materials and Methods section at the end of week 8 of the study. **(B)** The area under the curve (AUC) was calculated for ITT. Data are shown as means ± SEM (*n* = 11–12). ^*^p < 0.05 vs. LF group; ^#^*p* < 0.05 vs. HF group; *# vs. LF and HF groups. LF, low-fat diet-fed rats; HF, high-fat diet-fed rats; HF + 100, high-fat diet-fed rats treated with low dose ASE (100 mg/kg body weight); HF + 200, high-fat diet-fed rats treated with high dose ASE (200 mg/kg body weight).

To determine if ASE improves insulin sensitivity in HF diet-fed rats, we performed an IP ITT (Figure 3). The results showed that for all groups the difference in insulin sensitivity was not observed at 15 and 30 min after insulin injection (Figure 3A). After 1 h of insulin injection, no differences in blood glucose levels were observed between HF and LF groups. However, blood glucose levels for rats fed the low dose of ASE were significantly lower after insulin loading than HF diet-fed rats and LF diet-fed rats and remained lower than HF diet-fed rats after 2 h of injection. However, the high dose of ASE did not affect insulin sensitivity. The AUC (Figure 3B) for the ITT blood glucose levels showed that rats treated with the low-dose of ASE had significantly lower AUC than HF diet-fed rats. While the AUC for the high-dose ASE group did not differ from LF or HF groups.

### 3.4. Low-dose ASE treatment reduced pancreas weight and did not affect the degree of adiposity

**Figure 4:**
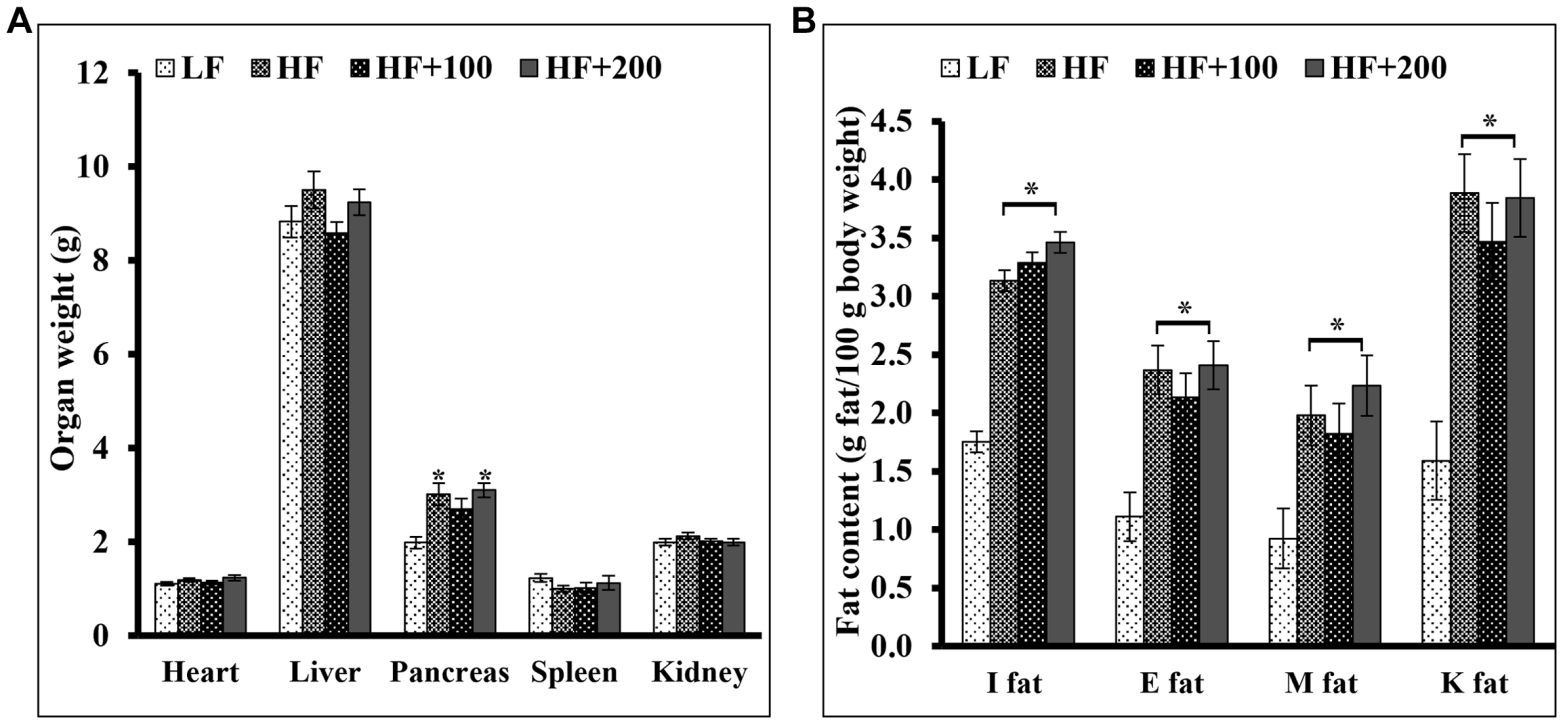
The ASE treatments for 9 weeks did not alter the fat content and the organs weight except the pancreas weight. At the end of the study, **(A)** the organs were isolated and weighed **(B)** subcutaneous fat pads [inguinal (I fat)] and several visceral fat pads [epididymal (E fat), retroperitoneal fat pads located on the kidneys (K fat), mesenteric (M fat)] were isolated and weighed. Data are shown as means ±SEM (*n* = 11–12). ^*^p < 0.05 vs. LF group. LF, low-fat diet-fed rats; HF, high-fat diet-fed rats; HF + 100, high-fat diet-fed rats treated with low dose ASE (100 mg/kg body weight); HF + 200, high-fat diet-fed rats treated with high dose ASE (200 mg/kg body weight); I fat, inguinal fat; E, epididymal fat; M, mesenteric fat; K, retroperitoneal fat pads located on the kidneys.

At the end of the treatment study, the organs (liver, heart, kidney, pancreas, spleen) were isolated and weighed (Figure 4A). No significant differences were observed in the weight of all organs except the pancreas weight between HF and LF groups. In addition, ASE did not affect the weight of the liver, heart, kidney, or spleen. The treatment with the high dose of ASE resulted in a significant increase in the pancreas weight compared to the LF group. The low dose of ASE reduced the effect of HF diet on the pancreas weight untreated and the high dose of ASE groups.

The body fat tissues such as subcutaneous pads (inguinal) and several visceral pads (epididymal, retroperitoneal fat pads located on the kidneys and mesenteric) were isolated. The weight and the percentage of inguinal and visceral fats were significantly heavier in HF diet-fed rats compared with the LF group (Figure 4B). Treatment with ASE did not affect the weight of any fat pad.

### 3.5. The ASE showed no sign of hepatotoxicity

At the end of the feeding study, the following parameters were measured after overnight fasting: body weight, serum insulin, FBG, total body fat calculated from inguinal and visceral fats, serum ALT and AST, the total lipid content in the liver (Table 1). The low-dose of ASE reduced HF diet-induced body weight gain. However, the high dose of ASE did not have the same effect. Treatment with both doses of ASE did not ameliorate HF diet-induced obesity rats.

**Table 1:** The metabolic parameter measured in the fasting state after 9 weeks of treatment.

Groups	LF	HF	HF + 100	HF + 200
Body weight (g)	331.7 ± 8.00^b*^	392 ± 11.64^a^	361.7 ± 9.67^ab^	380 ± 10.08^a^
Total fat (g/100 g body weight)	6.00 ± 0.50^b^	11.9 ± 0.93^a^	11.3 ± 0.91^a^	12.5 ± 0.61^a^
FBG (mg/dL)	68.1 ± 2.33^b^	81.2 ± 3.21^a^	70.6 ± 2.27^ab^	74.2 ± 5.07^ab^
Insulin (ug/L)	0.20 ± 0.044^b^	0.53 ± 0.087^ab^	0.59 ± 0.088^ab^	0.81 ± 0.184^a^
HOMA-IR	0.86 ± 0.19^b^	2.7 ± 0.50^ab^	2.5 ± 0.37^ab^	3.9 ± 1.14^a^
AST (U/L)	43.4 ± 6.42^a^	41.3 ± 5.67^a^	39.7 ± 2.76^a^	47.1 ± 2.43^a^
ALT (U/L)	26.3 ± 3.49^a^	27.3 ± 3.18^a^	33.3 ± 2.85^a^	29.0 ± 4.65^a^
Total liver lipids (mg/g wet liver)	213.9 ± 29^a^	346.8 ± 1.30^a^	289.0 ± 1.58^a^	245.3 ± 40.42^a^

^*^Means with different letters are significantly different (*p* < 0.05). Data are shown as Means ± SEM (*n* = 11–12). LF, low-fat diet-fed rats; HF, high-fat diet-fed rats; HF + 100, high-fat diet-fed rats treated with low dose ASE (100 mg/kg body weight); HF + 200, high-fat diet-fed rats treated with high dose ASE (200 mg/kg body weight); ALT, alanine aminotransferase; AST, aspartate aminotransferase.

Fasting insulin levels were measured and HOMA values were calculated. There were no significant differences in insulin levels or HOMA between HF and LF rats (Table 1). The low dose of ASE did not exert any effect on serum insulin or HOMA values compared to the HF group. While the serum insulin and value of HOMA values were significantly higher for rats treated with a high dose of ASE in comparison to the LF group.

Serum levels of ALT and AST, which are most frequently used to test liver function levels, did not differ between HF and LF groups (Table 1). In addition, the serum ALT and AST in rats were not affected by the oral administration of ASE. Total lipid content in the liver was evaluated (Table 1). Rats consuming the HF diet for 9 weeks had higher hepatic lipid contents compared to those in LF-fed rats, but this increase was not significant. There was a trend in reducing the liver lipid contents in the rats treated with ASE as compared to the HF group which was not significant.

### 3.6. ASE total phenolic and flavonoid content, and DPPH free radical antioxidant activity

**Figure 5:**
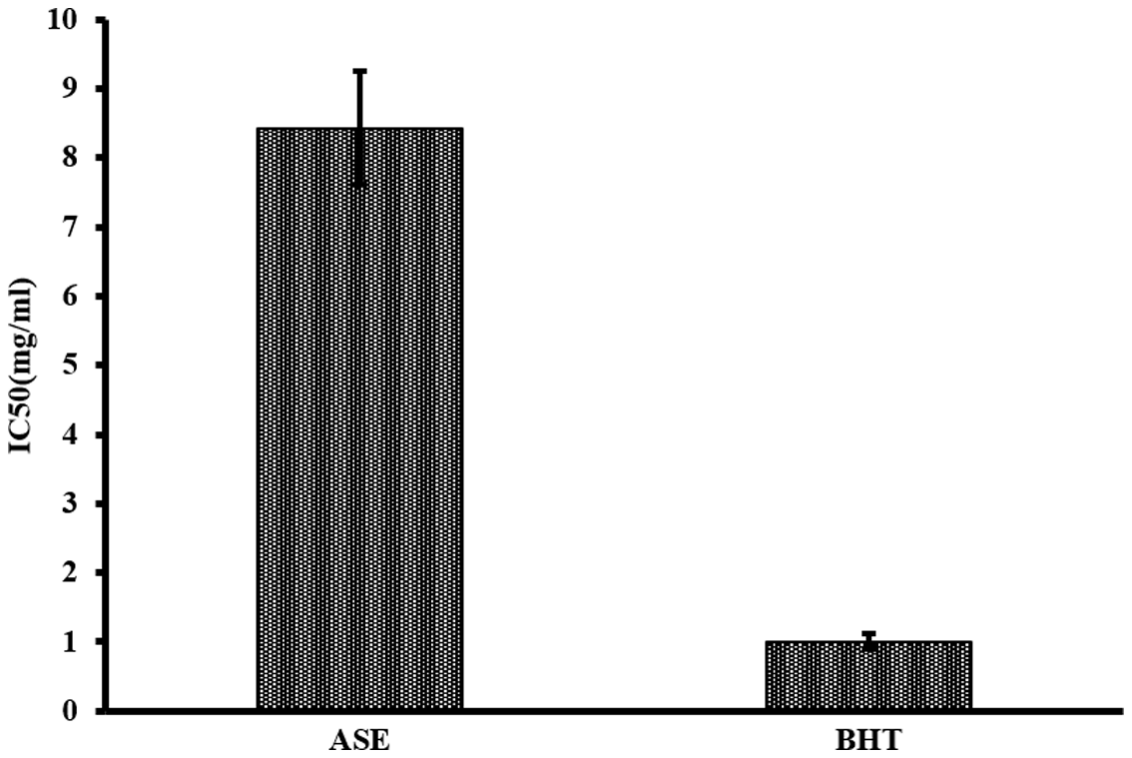
DPPH free radical antioxidant capacity of ASE compared to BHT.

The total phenolic content in a 5 mg/mL aqueous extract of *A. squamosa* leaves was 74.9 ± 0.491 mg of GAE per gram dry extract. The total flavonoid content in 5 mg/mL aqueous extract of *A. squamosa* leaves was 20.0 ± 0.091 mg QE per gram dry extract. The DPPH radical scavenging potency of *A. squamosa* leaves water extract was calculated as IC_50_ value, which was 8.43 ± 0.825 mg/mL. Results are shown in Figure 5.

## 4. Discussion

Several factors are documented to play a role in the etiology of obesity such as dietary factors, particularly the consumption of an HF diet rich in SFAs (29). It is well known that the HF diet increases weight gain, fat deposition, intrahepatic fat accumulation, impaired insulin sensitivity, and glucose tolerance in humans (30) and experimental animals such as Wistar rats (31). One of the reasonable molecular mechanisms to explain the ability of SFAs to the progression of obesity-induced IR. SFAs are associated with low rates of FA oxidation due to the notion that SFAs are less likely to activate the peroxisome proliferator-activated receptors, which is considered an important receptor for enhancing the degradation and mitochondrial β-oxidation pathways of FAs (32). Also, SFAs are more likely to be stored in adipose tissue and in non-adipose tissue such as liver and muscle than being used in energy production (12). This accumulation coexists with a dysregulation in the metabolism of the storage tissue. Our data demonstrated that HF diet (majorly composed of SFAs) caused a significant increase in total weight gain and significant expansion of adipose tissue mass since the HF diet-fed rats showed a large increase in visceral adipose tissue mass (perirenal, epididymal, mastering) and inguinal fat-pad weight as compared to rats fed the LF diet. No difference was observed in energy intake between HF and LF groups but the difference in weight gain can be explained mainly by the overconsumption of SFAs which is associated with increased weight gain more than other types of FAs (33).

The current results demonstrate for the first time a dose-dependent effect of administration ASE on body weight. In this study, rats treated with a low dose of ASE displayed lower body weight as compared with rats fed a HF diet during the study. While the high dose effect lasted for 6 weeks into the study. This effect could be due to the ability of a low dose of ASE to reduce appetite. Because when FI is viewed as cumulative intake, rats treated with the low dose of ASE showed significantly lower cumulative average daily FI as compared with rats in the other groups. This is in accordance with a previous study that demonstrated the effect of aqueous ASE on reducing the percentage of body weight change of STZ-induced diabetic rats after 28 days of treatments (19). Similarly, another study reported reduced food, energy and fluid intake for obese rats receiving hot-water ASE (250 mg/5 mL/kg) orally administered twice a day for 9 days (22).

Moreover, SFAs are associated with increased levels of several intracellular lipid metabolites particularly diacylglycerol (DAG) (9) and ceramides (34), products of SFAs metabolism and are purported to be involved in the pathogenesis of both hepatic IR (35) and peripheral IR (36) and they exert their effect by blocking the insulin-signaling pathway (37). The accumulation of DAG in the liver correlates with activation of stress kinases including JNK (38). JNK exerts its effect by blocking insulin-stimulated tyrosine phosphorylation of IRS-1 and IRS-2, which results in the inhibition of recruitment and activation of PI3-kinase (39) and subsequently limit the ability of insulin to activate glycogen synthase while increasing the gluconeogenesis (40). In addition, intracellular hepatic fat accumulation of SFAs may impair insulin-mediated suppression of hepatic glucose production resulting in the development of hyperglycemia (41) and glucose intolerance (42). Our results indicate that HF diet-induced hepatic IR, which led to the development of elevated FBG levels and impaired glucose tolerance is associated with a trend to increased intracellular hepatic fat accumulation. We further show for the first time that the treatment with a low dose of ASE for 9 weeks prevented hepatic IR as demonstrated by an improvement of FBG levels without hyperinsulinemia, and a significant decrease in blood glucose levels in response to IP insulin injection. Consistently, the aqueous leaves extract has been reported to significantly reduce FBG levels in STZ-induced diabetic rats when giving at a dose of 350 mg/kg (19). Additionally, obese rats orally fed hot-water ASE from leaves (250 mg/5 mL/kg) twice a day for 9 days had improved glucose tolerance and plasma insulin response which was accompanied by an inhibition of Dipeptidyl Peptidase (DPP)-IV enzyme activity suggesting improved insulin action (22). DPP-IV inhibition is considered one of the promising targets for treating T2DM as it increases the levels of incretin hormones such as Glucagon-Like Peptide-1 (GLP-1) and Glucose-dependent insulinotropic-polypeptide (GIP) which promote glucose-stimulated insulin release (43). *In vitro*, aqueous ASE from leaves inhibited DPP-IV enzyme activity by directly acting and blocking the active binding site (22).

The excessive formation of ceramide in non-adipose tissue such as skeletal muscle may induce peripheral IR (44). The ceramide is a potent antagonist of insulin action due to its activation of Toll-like receptor 4, which is essential for mounting inflammatory responses associated with innate immunity (45). The TLR 4 activation induces IκB kinase-mediated proinflammatory molecule production, leading to the inhibition of insulin stimulation of the serine/threonine kinase Akt/PKB resulting in inhibition of Glucose Transporter Type 4 in the skeletal muscle (44). This inhibition in glucose uptake results in a compensatory hyperinsulinemia state without developing fasting hyperglycemia. The price paid to prevent the development of fasting hyperglycemia in peripheral IR is the effect on tissues that retain normal insulin sensitivity including the liver and pancreas. The compensatory hyperinsulinemia state may induce more FFAs to enter the liver for hepatic TG synthesis and increased hepatic VLDL-TG secretion, which increases FFAs in plasma and the development of hypertriglyceridemia without hepatic fat accumulation (46).

Our finding suggests that the effect of a low dose of ASE was greater on FI, body weight gain, and insulin sensitivity than a high dose of ASE. This might be explained by a decreased daily FI in rats fed the low dose of ASE. Phenolic compounds, including flavonoids, are known to exert antidiabetic, antioxidant, anti-inflammatory, and other therapeutic effects (47). The bioavailability of these compounds varies significantly from one plant to another (48). In this study, the total phenolic content of the ASE was evaluated, which was 74.9 ± 0.491 GAE mg/g. The results agreed with a previous study showing a total phenolic content of 75.8 ± 1.31 GAE mg/g derived from the ethanolic extract of dried leaves powder (49). Another study indicated a very high phenolic content in aqueous extract 210 GAE mg/g, but the content was higher in acetone, methanolic, and ethanolic extracts (50). Likewise, phenolic content in methanolic *A. squamosa* leaves extract in Lebanon (117.2 GAE mg/g) (51), and Sudan (93.6 GAE mg/g) (52) was very high compared to its content in the present study. Other studies showed that the total phenolic content was high in *A. squamosa* leaves wherein ethanol was used as an extraction solvent in India (264 GAE mg/g), and Lebanon (112.9 GAE mg/g) (51). The current study also evaluated the flavonoid content of *A. squamosa* which was 20.032 ± 0.091 QE mg/g. Similar content was obtained from dried leaves of ethanolic *A. squamosa* extract in India (49). Our results indicated a higher flavonoid content in *A. squamosa* extract compared to another study in Saudi Arabia, where a minimal amount of flavonoids were found in both aqueous and methanolic plant extract (53). Another study indicated that the leaves’ methanolic extract yielded around 223 QE mg/g of total flavonoids, which is very high compared to our results (52). Hot-water ASE from leaves analysis by HPLC procedure showed that it contains flavonoids such as rutin, isoquercitrin and quercetin (22). It is reasonable to explain the absence of the beneficial effects of high dose ASE by a possible presence of compounds that could act adversely at high levels. It has been reported that high doses of flavonoids may inhibit key proteins in hormone metabolism and cell function (54). Therefore, future studies are needed to investigate the effect of compounds present in ASE at different doses on glucose control and metabolism. The LD50 dose of water extract was found to be more than 5 g/kg body weight, and no deaths were observed (19). In the present study, we also show that ASE at tested doses had no characteristic hepatotoxicity in rats, suggesting that it is likely safe for its use as a dietary supplement.

*Annona squamosa* extract, in our study, had a free radical scavenging activity of IC_50_ = 8.43 ± 0.825 mg/mL, indicating a lower antioxidant capacity than methanolic (IC_50_ = 0.01361 mg/mL) and ethanolic extracts of the plant (IC_50_ = 0.01597 mg/mL) (51). Mariod and colleagues indicated that the methanolic extract had a free radical scavenging activity of IC_50_ = 0.00781 mg/mL (52). Although the methanolic extract had a higher antioxidant potency, several studies indicated the high antioxidant activity of the aqueous *A. squamosa* extract compared to results found in our study (17, 55, 56). The IC_50_ of ASE in our study is considered high compared to the standard antioxidant BHT (IC_50_ = 1.01 ± 0.11 mg/mL). This low antioxidant capacity could be associated with the plant extraction method. The wide variations in the extracts’ content of total phenols, flavonoids, and their antioxidant capacity might be affected by the environment, the seasonal variations, and the plants’ handling procedure (48).

In summary, we provide evidence that short-term administration of ASE may be beneficial in ameliorating HF-diet induced obesity and IR. In the long-term, only the low dose of ASE (100 mg/kg/day) was able to decrease HF diet-induced body weight gain in rats, which was associated with a reduction in cumulative food and calorie intake. In addition, the low dose of ASE improved insulin sensitivity and reduced FBG levels, thereby decreasing the HF diet-induced metabolic abnormalities. Our results indicate that the high dose of ASE may be associated with the development of fasting hyperinsulinemia without hyperglycemia concomitantly with enlargement of the pancreas. These changes may be responsible for the development of peripheral IR. Therefore, our findings demonstrate that lower doses of ASE maybe be used as a dietary supplement to prevent obesity-induced IR and related metabolic disorders. Given that obesity and IR are risk factors for developing T2D, ASE could be capable of preventing the pathogenesis of T2D and it may have the potential to prevent diabetes. However, our results should not be generalized and should be used carefully. Animal models, rats in this study, might be cost-effective and efficient experiments for understanding human behavior and designing new therapies for several diseases, but they are not identical to the human body. Also, animal studies could be limited by the environmental factors affecting rats’ behaviors, such as housing conditions, researcher skills, and room temperature (57). Hence, further studies are needed to elucidate the molecular mechanisms underlying the beneficial metabolic effects of ASE, and future clinical trials that can confirm the therapeutic effects of ASE on obesity and diabetes are needed.

## Data availability statement

The original contributions presented in the study are included in the article/Supplementary material, further inquiries can be directed to the corresponding author.

## Ethics statement

The animal study was reviewed and approved by Animal Care and Use Committee at the Jordan University of Science and Technology (362-2018).

## Author contributions

HA and DL: conceptualization. HA, DL, and RM: methodology. RM: formal analysis, investigation, writing—original draft preparation, and visualization. HA and AA-N: resources, supervision, project administration, and funding acquisition. RM and HA: data curation. HA, AA-N, and DL: writing—review and editing. All authors contributed to the article and approved the submitted version.

## Funding

This research was funded by Deanship of Research at Jordan University of Science and Technology grant number 20180385.

## Conflict of interest

The authors declare that the research was conducted in the absence of any commercial or financial relationships that could be construed as a potential conflict of interest.

## Publisher’s note

All claims expressed in this article are solely those of the authors and do not necessarily represent those of their affiliated organizations, or those of the publisher, the editors and the reviewers. Any product that may be evaluated in this article, or claim that may be made by its manufacturer, is not guaranteed or endorsed by the publisher.

## Supplementary material

SUPPLEMENTARY FIGURE S1 ASE treatment effect on body weight and food and caloric intake. **(A)** The percentage change in body weight was the percent difference between weights at the beginning and end of the study. **(B)** Food intake was recorded weekly. **(C)** Energy consumption was calculated based on average daily energy consumption (kcal) for each rat. Data are shown as means ± SEM (*n* =11–12). **p* < 0.05 vs. LF group; #*p* < 0.05 vs. HF group; ## *p* < 0.05 vs. HF+100 group. LF: low-fat diet-fed rats; HF: high-fat diet-fed rats; HF+100: high-fat diet-fed rats treated with low dose ASE (100 mg/kg body weight); HF+200: high- fat diet-fed rats treated with high dose ASE (200 mg/kg body weight).
