# Supplementary figures and images for: Low-dose of phenolic rich extract from Annona squamosa Linn leaves ameliorates insulin sensitivity and reduces body weight gain in HF diet-induced obesity
Source: Front Nutr. 2023 Jul 19;10:1146021. doi: 10.3389/fnut.2023.1146021 (PMC10394232; doi:10.3389/fnut.2023.1146021)

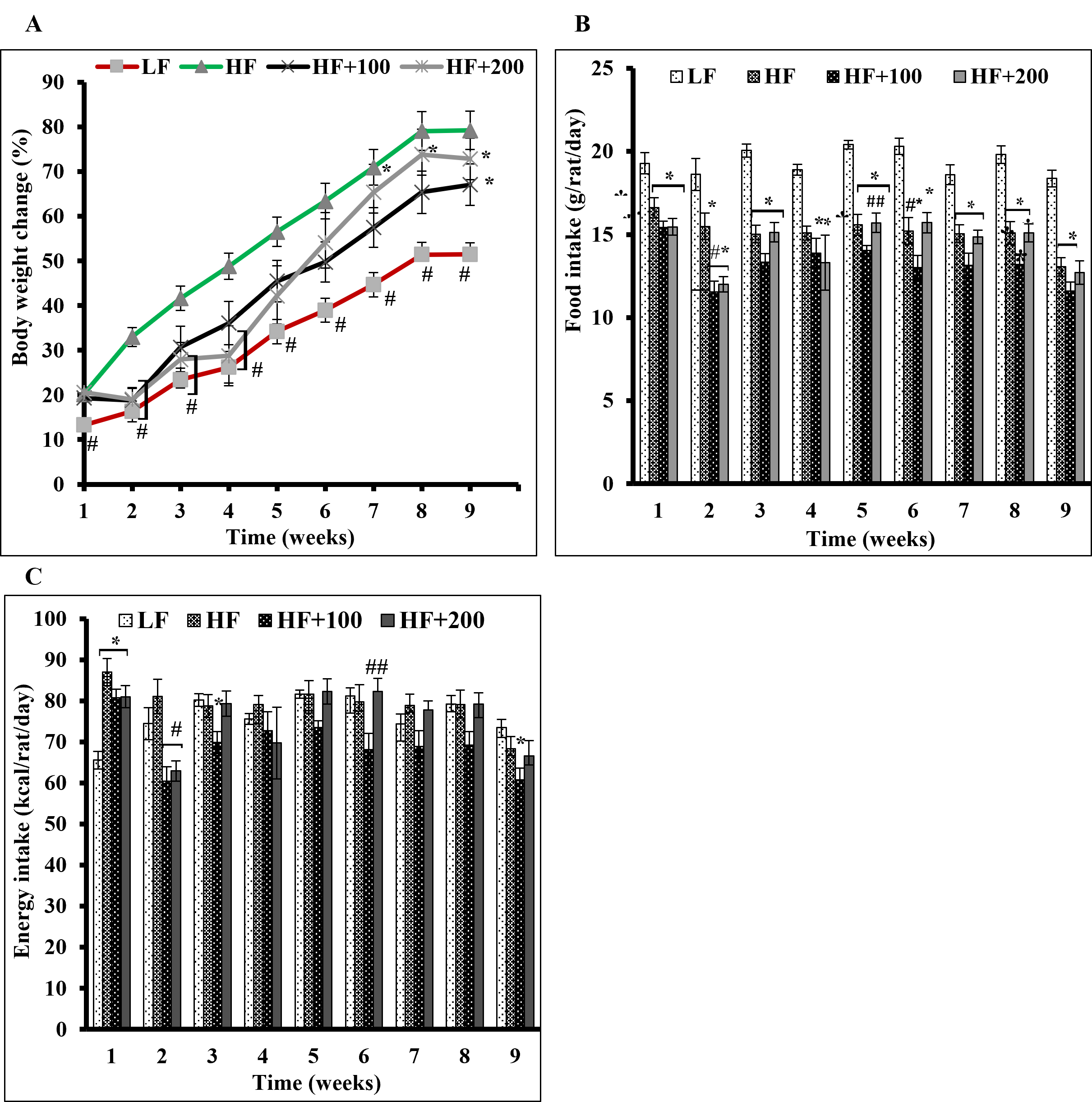

Supplement: SUPPLEMENTARY FIGURE S1 — ASE treatment effect on body weight and food and caloric intake. (A) The percentage change in body weight was the percent difference between weights at the beginning and end of the study. (B) Food intake was recorded weekly. (C) Energy consumption was calculated based on average daily energy consumption (kcal) for each rat. Data are shown as means ± SEM (n =11–12). *p < 0.05 vs. LF group; #p < 0.05 vs. HF group; ## p < 0.05 vs. HF+100 group. LF: low-fat diet-fed rats; HF: high-fat diet-fed rats; HF+100: high-fat diet-fed rats treated with low dose ASE (100 mg/kg body weight); HF+200: high- fat diet-fed rats treated with high dose ASE (200 mg/kg body weight). [file Image_1.TIF]
