# Supplementary material for: Low-dose of phenolic rich extract from Annona squamosa Linn leaves ameliorates insulin sensitivity and reduces body weight gain in HF diet-induced obesity
Source: Front Nutr. 2023 Jul 19;10:1146021. doi: 10.3389/fnut.2023.1146021 (PMC10394232; doi:10.3389/fnut.2023.1146021)
Supplement: Supplementary file 2 [file Table_1.DOCX]

**Supplementary Table S1. Diet composition.**

|  | **Low-fat diet** | **High-fat diet** |
| --- | --- | --- |
| **Macronutrients (g/kg)** | | |
| Fat | 18.0 | 347.3 |
| Protein | 144.5 | 260.5 |
| Carbohydrates | 663.0 | 260.5 |
| **Minerals (g/kg)** | | |
| Potassium Citrate, Monohydrate | 21.45 | |
| Calcium Phosphate, Dibasic | 16.9 | |
| Calcium Carbonate, Light, USP | 7.15 | |
| Sodium Chloride | 3.367 | |
| Magnesium Sulfate, Heptahydrate | 3.3475 | |
| Magnesium Oxide, Heavy, DC USP | 0.5447 | |
| Ferric Citrate | 0.273 | |
| Manganese Carbonate Hydrate | 0.15925 | |
| Zinc Carbonate | 0.0728 | |
| Chromium Potassium Sulfate | 0.02535 | |
| Copper Carbonate | 0.01365 | |
| Ammonium Molybdate Tetrahydrate | 0.0039 | |
| Sodium Fluoride | 0.0026 | |
| Sodium Selenite | 0.00065 | |
| Potassium Iodate | 0.00065 | |
| **Vitamins (g/kg)** | | |
| Vitamin E Acetate, 50% | 0.129 | |
| Niacin | 0.0387 | |
| Biotin, 1% | 0.0258 | |
| Pantothenic Acid, d, Calcium | 0.02064 | |
| Vitamin D3, 100,000 IU/g | 0.0129 | |
| Vitamin B12, 0.1% Mannitol | 0.0129 | |
| Vitamin A Acetate, 500,000 IU/g | 0.01032 | |
| Pyridoxine HCl | 0.00903 | |
| Riboflavin | 0.00774 | |
| Thiamine HCl | 0.00774 | |
| Folic Acid | 0.00258 | |
| Menadione Sodium Bisulfite | 0.001032 | |
